# Supplementary material for: Modeling glioblastoma heterogeneity as a dynamic network of cell states
Source: Mol Syst Biol. 2021 Sep 16;17(9):e10105. doi: 10.15252/msb.202010105 (PMC8444284; doi:10.15252/msb.202010105)
Supplement: Supplementary file 6 — Source Data for Figure 5 [file MSB-17-e10105-s004.zip › Figure5A_sourcedata/GSEA_3071/nosigdb_stateIII.GseaPreranked.1622795155562/RICHARDS_ZHONG_NATURE_2018_OPC_UPREG.html]

Details for gene set RICHARDS\_ZHONG\_NATURE\_2018\_OPC\_UPREG[GSEA]

|  || Dataset | state33071 |
| Phenotype | NoPhenotypeAvailable |
| Upregulated in class | na\_neg |
| GeneSet | RICHARDS\_ZHONG\_NATURE\_2018\_OPC\_UPREG |
| Enrichment Score (ES) | -0.6289455 |
| Normalized Enrichment Score (NES) | -3.2411325 |
| Nominal p-value | 0.0 |
| FDR q-value | 0.0 |
| FWER p-Value | 0.0 |
Table: GSEA Results Summary

  

Fig 1: Enrichment plot: RICHARDS\_ZHONG\_NATURE\_2018\_OPC\_UPREG      
 Profile of the Running ES Score & Positions of GeneSet Members on the Rank Ordered List

  

| PROBE | GENE SYMBOL | GENE\_TITLE | RANK IN GENE LIST | RANK METRIC SCORE | RUNNING ES | CORE ENRICHMENT || 1 | RHOC |  |  | 92 | 0.414 | -0.0067 | No |
| 2 | ETV5 |  |  | 818 | -0.270 | -0.1820 | No |
| 3 | BCHE |  |  | 867 | -0.272 | -0.1832 | No |
| 4 | SPATA6 |  |  | 1225 | -0.291 | -0.2630 | No |
| 5 | FIBIN |  |  | 1458 | -0.304 | -0.3102 | No |
| 6 | ETV1 |  |  | 1495 | -0.306 | -0.3069 | No |
| 7 | SLITRK2 |  |  | 1725 | -0.320 | -0.3527 | No |
| 8 | SPRY4 |  |  | 1970 | -0.334 | -0.4018 | No |
| 9 | PLEKHB1 |  |  | 2032 | -0.337 | -0.4036 | No |
| 10 | TRIB2 |  |  | 2105 | -0.342 | -0.4081 | No |
| 11 | SCD5 |  |  | 2128 | -0.344 | -0.3996 | No |
| 12 | TTYH1 |  |  | 2397 | -0.365 | -0.4536 | No |
| 13 | MMP2 |  |  | 2527 | -0.375 | -0.4714 | No |
| 14 | ALCAM |  |  | 2853 | -0.403 | -0.5384 | No |
| 15 | PDE4B |  |  | 2878 | -0.406 | -0.5279 | No |
| 16 | ITGB8 |  |  | 3026 | -0.424 | -0.5483 | No |
| 17 | SEMA5A |  |  | 3229 | -0.447 | -0.5819 | No |
| 18 | CD63 |  |  | 3258 | -0.452 | -0.5706 | No |
| 19 | OLIG1 |  |  | 3486 | -0.503 | -0.6083 | Yes |
| 20 | LRRC4C |  |  | 3512 | -0.512 | -0.5937 | Yes |
| 21 | TSPAN7 |  |  | 3528 | -0.520 | -0.5762 | Yes |
| 22 | CD82 |  |  | 3569 | -0.534 | -0.5646 | Yes |
| 23 | SERPINE2 |  |  | 3573 | -0.537 | -0.5433 | Yes |
| 24 | PLAT |  |  | 3600 | -0.547 | -0.5275 | Yes |
| 25 | SPRY1 |  |  | 3605 | -0.552 | -0.5059 | Yes |
| 26 | LSAMP |  |  | 3623 | -0.561 | -0.4872 | Yes |
| 27 | CD9 |  |  | 3634 | -0.567 | -0.4665 | Yes |
| 28 | DUSP6 |  |  | 3648 | -0.577 | -0.4461 | Yes |
| 29 | RFTN2 |  |  | 3654 | -0.582 | -0.4235 | Yes |
| 30 | SOX6 |  |  | 3675 | -0.596 | -0.4042 | Yes |
| 31 | GNG7 |  |  | 3722 | -0.633 | -0.3900 | Yes |
| 32 | SLC35F1 |  |  | 3724 | -0.635 | -0.3642 | Yes |
| 33 | LIMA1 |  |  | 3774 | -0.706 | -0.3479 | Yes |
| 34 | SOX8 |  |  | 3797 | -0.737 | -0.3233 | Yes |
| 35 | SPARC |  |  | 3811 | -0.759 | -0.2955 | Yes |
| 36 | S100B |  |  | 3853 | -0.845 | -0.2713 | Yes |
| 37 | SCRG1 |  |  | 3869 | -0.904 | -0.2380 | Yes |
| 38 | PMP2 |  |  | 3876 | -0.959 | -0.2002 | Yes |
| 39 | PTN |  |  | 3884 | -1.022 | -0.1601 | Yes |
| 40 | GPM6B |  |  | 3889 | -1.047 | -0.1181 | Yes |
| 41 | PTPRZ1 |  |  | 3913 | -1.342 | -0.0689 | Yes |
| 42 | PDGFRA |  |  | 3928 | -1.773 | 0.0003 | Yes |
Table: GSEA details [plain text format]

  

Fig 2: RICHARDS\_ZHONG\_NATURE\_2018\_OPC\_UPREG: Random ES distribution      
 Gene set null distribution of ES for **RICHARDS\_ZHONG\_NATURE\_2018\_OPC\_UPREG**
